# Impact of COVID-19 Outbreak on Emergency Visits and Emergency Consultations: A Cross-Sectional Study

**DOI:** 10.7759/cureus.14052

**Published:** 2021-03-23

**Authors:** Buğra İlhan, Göksu Bozdereli Berikol, Halil Dogan

**Affiliations:** 1 Emergency, Bakırköy Dr. Sadi Konuk Training and Research Hospital, İstanbul, TUR

**Keywords:** covid-19, pandemics, emergency visits, emergency consultations

## Abstract

Background

This study aimed to determine the effect of the COVID-19 outbreak on emergency department (ED) visits and emergency consultations according to the triage levels indicating the patients' urgency.

Methods

A cross-sectional retrospective study was performed in the ED of a tertiary training and research hospital between 1 April and 31 May 2020 in İstanbul, Turkey. The daily count of emergency visits and the count of the emergency consultations during the study period were recorded. The emergency visits and consultations in the same months of the previous year (1 April-31 May 2019) were included as a control group.

Results

Approximately 50% reduction in ED visits and a 30% reduction in emergency consultations were detected. A significant decrease was detected in all triage levels of visits and emergency consultations (p < 0.001). Within total ED visits, a significant increase was found in the red (4.32% vs. 4.74%) and yellow (21.66% vs. 33.16%) triage levels visit rates, while the green (74.01% vs. 62.1%) level was decreased. Within total emergency consultations, anesthesiology (0.83% vs. 1.56%) and cardiology (3.17% vs. 3.75%) consultation rates increased, neurology (2.22% vs. 1.15%), orthopedics (3.53% vs. 3.01%), and ophthalmology (2.89% vs. 1.57%) consultation rates decreased, internal medicine (2.45% vs. 2.49%), and general surgery (4.46% vs. 4.64%) consultation rates did not change.

Conclusions

During the COVID-19 pandemic, ED visits at all triage levels decreased. While the rate of critical patient visits increased, non-emergency patient visit rates decreased. The total count of consultations decreased, while the total consultation rates increased. The management of the COVID-19 pandemic will be easier by using or developing appropriate triage scores, as well as establishing good interdisciplinary coordination.

## Introduction

In December 2019, after the cases of pneumonia of unknown cause began to appear in Wuhan, China, a new SARS CoV-2 was detected as a causative agent. This virus, which started to spread rapidly afterward, was named COVID-19 and was declared a pandemic by the World Health Organization (WHO) [1]. So far, more than 110 million confirmed cases and over 2,500,000 deaths have been reported [2]. There have been many studies in the literature on the main symptoms, treatment, and prevention methods of this disease, which transmits from person to person, and causes severe damage to many systems, especially the respiratory system [3]. Research continues on this disease, which does not have a proven, reliable treatment.

With the COVID-19 outbreak, many countries have taken severe and swift measures to prevent the disease’s spread and protect their citizens. In this process, COVID-19 patients became a priority, and all arrangements were made for the appropriate management of COVID-19 patients. This change brought along some problems. The most crucial issue has been the indirect effect of the COVID-19 outbreak on potentially life-threatening conditions [4]. During the pandemic period, the decrease in the number of referrals of patients with acute coronary syndrome and acute stroke was noted [5-7]. A similar reduction was found in the admissions of trauma patients [8].

Emergency departments (EDs) have been the site of first presentation of emergency patients during the pandemic period as in the pre-pandemic period. In our country, all presentations of COVID-19 suspected patients were made to EDs until pandemic outpatient clinics were become functional. After COVID-19 outpatient clinics were put into use, EDs continued to serve non-critical patients in addition to critically ill patients (both COVID and non-COVID). During the COVID-19 outbreak, COVID and non-COVID patients continued to receive care from EDs.

During the pandemic period, there have been changes in ED visits as in other clinics. However, there are limited studies in the literature evaluating the changes in ED visits. Also, there are no studies on the COVID-19 outbreak’s impact on critical and non-critical patient visits and emergency consultations together. This study aims to determine the effect of the COVID-19 outbreak on ED visits and emergency consultations according to the triage levels.

## Materials and methods

Study design

This retrospective observational study was conducted in the ED of a tertiary training and research hospital in Turkey’s most populous city, İstanbul. Approximately 350,000 patients present annually to the ED, where the study was conducted. It is the primary center for percutaneous coronary intervention and thrombolytic therapy for cardiac and neurological emergencies. Additionally, it is a level three trauma center and a referral center for oncological emergencies.

The Ministry of Health assigned the hospital a “pandemic hospital” for COVID-19 suspected patients after the first COVID-19 case in the country was diagnosed in the second week of March. It serves approximately 1000 new COVID-19 suspected patients daily with all clinics, wards, intensive care units, and ED. Additionally, non-COVID-19 patients continued to take care from the hospital. The study started after the approval of the local ethics committee (Approval ID: 2020/365).

Patient records

**Figure 1:**
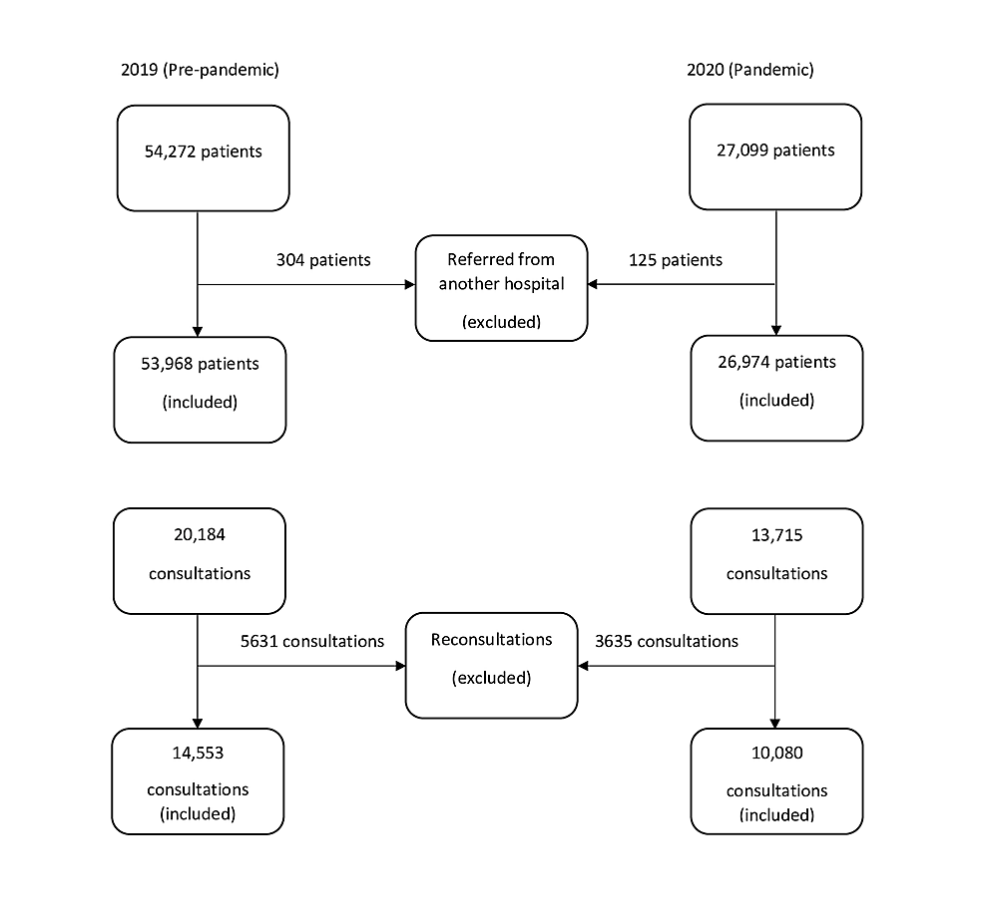
Flow diagram of the study

Emergency patient data, recorded between April 1, 2020 and May 31, 2020, were included in the study. The daily count of ED visits during the study period and the count of the consultations requested from ED were recorded. The data were obtained from electronic medical records. The patient visits and consultations in the same months of the previous year (April 1, 2019 - May 31, 2019) were included as a control group to reflect the pre-pandemic period and to make the comparison reliable. Patients referred from another medical center and consultations requested from the same department (reconsultations) were excluded from the study. In total, there were 26,974 patients and 10,080 consultations in the pandemic group and 53,968 patients and 14,553 consultations in the pre-pandemic (control) group. The flow diagram of the study is shown in Figure 1.

Definitions and groups

Patients were triaged to red, yellow and green areas by a registered triage nurse who had previously received triage training organized by the Health Ministry.

Red Color

Life-threatening situations require rapid (within minutes), aggressive approach, and immediate, simultaneous evaluation/treatment.

Yellow Color

Cases that have the possibility of a life-threatening condition, risk of limb loss, and significant morbidity. Conditions with middle and long-period symptoms and potential of severity (Patients with abnormal respiratory rate, pulse, blood pressure, oxygen saturation, body temperature; patients who need medical treatment, and patients with a subjective pain score of 80% of the maximum score).

Green Color

The patients whose general condition is stable and who can be treated on an outpatient basis. Patients with simple health problems were not at risk of morbidity or life-threatening situation by waiting for 1-4 hours.

Examples of cases used in triage color coding are given in Appendix 1.

Patient examination and consultation rate

All patients were first evaluated in the triage area and were transferred to areas (red, yellow, or green area) suitable for their severity levels. The researchers calculated the examination and consultation rates of patients who presented to these areas according to the total count of patient examinations to the ED during the pandemic and pre-pandemic period. The total count of examinations represented the total patients’ count seen by an emergency physician in the ED. Leaving without being seen (LWBS) count was not included in the calculations not to affect the results. The patient examination and consultation rates were calculated according to the following formula:

Patient examination rate = Count of patients / Total count of examinations x 100

Patient consultation rate = Count of consultations / Total count of examinations x 100

Count of patients: Patient counts which were presented to the ED red, yellow, and green areas.

Count of consultations: Consultation counts requested from ED red, yellow, and green areas.

Total count of examinations: Total count of patients who were seen by an emergency physician (Total count of ED visits - Leaving without being seen count).

Outcomes

The study’s primary outcome is the change in emergency visits according to the triage levels during the COVID-19 outbreak. Secondary outcomes are the change in the total emergency visits during the COVID-19 outbreak, the frequency of consultations requested from the ED, and the count of LWBS patients.

Data analyses

Numerical variables were represented as mean ± standard deviation or median (interquartile range). Categorical variables were presented as absolute values and percentages. Kolmogorov-Smirnov and Shapiro-Wilk tests were used to evaluate the distributions. Independent groups were assessed using the independent t-test and the Mann-Whitney U test. The relationship between categorical variables was assessed using the chi-square test. Spearman or Pearson test was used in correlation analysis according to the distribution of the data. The statistical significance level was set as p < 0.05. Statistical analysis of the data was performed with IBM SPSS version 23 program (IBM Corp., Armonk, NY).

## Results

During the study period, 53,968 emergency visits were recorded between April and May 2019, while 26,974 patients were recorded in the same months of 2020. Approximately 50% reduction in total ED visits and 30% reduction in emergency consultations were detected. ED visits and consultations requested from ED are shown in Table 1.

**Table 1:** Emergency visits and consultations before and after COVID-19 outbreak * Total count of patients who were seen by an emergency physician **Total consultation count requested from emergency department

	Pre-pandemic (n)	Pandemic (n)
Total emergency visits	53,968	26,974
Leaving without being seen	2,436	1,473
Total examinations*	51,532	25,501
Triage level/area		
Red	2,228	1,209
Yellow	11,163	8,456
Green	38,141	15,836
Total consultation count**	14,553	10,080
Red	3,199	2,026
Yellow	10,651	7,135
Green	702	919
Consultation counts of clinics		
Anesthesiology	426	397
Cardiology	1635	956
Neurology	1142	292
Infectious disease	387	3571
Internal medicine	1264	634
Gastroenterology	445	230
Pulmonology	155	5
General surgery	2299	1184
Orthopedics	1821	768
Ophthalmology	1487	401
Otolaryngology	847	322
Thoracic surgery	285	183
Neurosurgery	394	168
Obstetrics and gynecology	1068	471
Cardiovascular surgery	370	197
Urology	348	218
Plastic surgery	37	11
Dermatology	102	13
Interventional radiology	15	11
Forensic science	3	0
Psychiatry	39	5

A significant decrease was detected in all triage levels of visits during the COVID-19 outbreak (p < 0.001). Consultation counts in the red and yellow triage levels were significantly decreased (p < 0.001). However, there was no significant difference in the consultation counts in the green triage level (p = 0.967). While the count of consultations for all other clinics decreased or did not change, a significant increase was found in the count of infectious diseases consultations requested from ED (p < 0.001). All LWBS patients were at the green triage level in both pandemic and pre-pandemic groups. The changes in emergency visits and emergency consultations are shown in Table 2.

**Table 2:** The changes in emergency visits and emergency consultations before and after COVID-19 outbreak ^ a ^median (IQR: Interquartile range), ^b ^mean ± standard deviation, * Mann-Whitney U test, ** Independent t-test, ^ᶲ ^Total count of patients who were seen by an emergency physician, ^ᶲᶲ^ Total consultation count requested from emergency department, p < 0.05 considered significant. LWBS: Leaving without being seen, IR: Interventional radiology, OB-GYN: Obstetrics and gynecology, CVS: Cardiovascular surgery.

	Pre-pandemic	Pandemic	p
Total emergency visits	877 (835.5-919.5)^a^	444 (366.5-506)^a^	<0.001*
Total examinationsᶲ	844.79±59.49^b^	418.05±93.86^b^	<0.001*
Triage level/area			
Red	36.52±5.31^b^	19.82±4.47^b^	<0.001*
Yellow	183±19.06^b^	138.62±35.48^b^	<0.001*
Green	616 (583-680)^a^	230 (180-323.5)^a^	<0.001*
LWBS	39 (24.5-51)^a^	21 (14-31)^a^	<0.001*
Total consultationsᶲᶲ	238.57±23.41^b^	165.25±29.66^b^	<0.001**
Red	50 (44-60.5)^a^	32 (27-38.5)^a^	<0.001**
Yellow	174.61±20.26^b^	116.97±28.21^b^	<0.001**
Green	11 (8-14.5)^a^	11 (0-25)^a^	0.967*
Consultations			
Anesthesiology	7 (5-8)^a^	6 (4-8)^a^	0.218*
Cardiology	26.8±6.99^b^	15.6±5.81^b^	<0.001**
Neurology	19 (17-22)^a^	4 (3-6)^a^	<0.001*
Infectious disease	6 (5-8)^a^	49 (36-84)^a^	<0.001*
Internal medicine	20.72±5.4^b^	10.3±4.14^b^	<0.001**
Gastroenterology	8 (5.5-9)^a^	3 (2-5)^a^	<0.001
Pulmonology	2 (0-5)^a^	0 (0-0)^a^	<0.001*
General surgery	37.69±7.04^b^	19.41±8.16^b^	<0.001**
Orthopedics	31 (24-34)^a^	12 (7-17.5)^a^	<0.001*
Ophthalmology	24 (20-28)^a^	6 (3-10.5)^a^	<0.001*
Otolaryngology	14 (11-17)^a^	5 (2-8)^a^	<0.001*
Thoracic surgery	5 (3-6)^a^	3 (1.5-4)^a^	0.297*
Neurosurgery	7 (5-8)^a^	2 (1-4)^a^	<0.001*
OB-GYN	17.51±4.27^b^	7.72±3.46^b^	<0.001**
CVS	6 (4-7)^a^	3 (2-5)^a^	<0.001*
Urology	6 (4-7)^a^	3 (1-5)^a^	<0.001*
Plastic surgery	0 (0-1)^a^	0 (0-0)^a^	0.003*
Dermatology	1 (1-2.5)^a^	0 (0-0)^a^	<0.001*
IR	0 (0-0)^a^	0 (0-0)^a^	0.297*
Forensic science	0 (0-0)^a^	0 (0-0)^a^	0.081*
Psychiatry	0 (0-1)^a^	0 (0-0)^a^	<0.001*

A moderate-strong correlation was found between the total count of ED examinations, the count of patient visits at all triage levels, and LWBS patients’ count. The correlations between the total count of examinations, the count of emergency visits, and consultation counts are shown in Table 3.

**Table 3:** Correlations of total examination counts, emergency visits, and emergency consultation counts ^ᶲ^Total count of patients who were seen by an emergency physician, ^ᶲᶲ^Total consultation count requested from emergency department, *Spearman correlation coefficient, p < 0.05 considered significant LWBS: Leaving without being seen

	Emergency visits					Consultation countsᶲᶲ			
	Total examinationsᶲ	Red	Yellow	Green	LWBS	Total consultations	Red	Yellow	Green
Total examinationsᶲ	1.000*	0.752*	0.645*	0.982*	0.628*	0.820*	0.652*	0.771*	0.103*
p	.	<0.001	<0.001	<0.001	<0.001	<0.001	<0.001	<0.001	0.260

**Figure 2:**
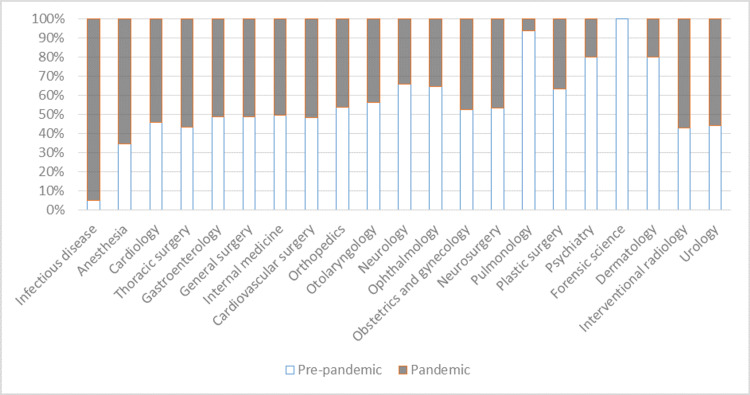
The changes in the consultation rates of the clinics before and after COVID-19 outbreak

When the patient examination rates were evaluated, a significant increase was found in the red and yellow triage levels, while a significant decrease was found at the green triage level. A significant increase was found in the consultation rates requested from all triage levels. According to the consulting departments, a significant increase was found in consultation rates of infectious diseases, anesthesiology, and cardiology departments (p <0.001). There was a significant decrease in consultation rates of the neurology department (p <0.001). The changes in examination and consultation rates are shown in Table 4. The changes in the consultation rates of the clinics are shown in Figure 2.

**Table 4:** The changes in patient examination and consultation rates before and after COVID-19 outbreak Patient examination rate = Count of patients / Total count of examinations x 100 Patient consultation rate = Count of consultations / Total count of examinations x 100 *Chi-square test, p < 0.05 considered significant

	Pre-pandemic (%)	Pandemic (%)	p
Triage level/area			
Red	4.32	4.74	0.008*
Yellow	21.66	33.16	<0.001*
Green	74.01	62.1	<0.001*
Leaving without being seen	4.73	5.78	<0.001*
Total consultation rate	28.24	39.53	<0.001*
Red	6.21	7.94	<0.001*
Yellow	20.67	27.98	<0.001*
Green	1.36	3.6	<0.001*
Consultations			
Anesthesiology	0.83	1.56	<0.001*
Cardiology	3.17	3.75	<0.001*
Neurology	2.22	1.15	<0.001*
Infectious disease	0.75	14	<0.001*
Internal medicine	2.45	2.49	0.779*
Gastroenterology	0.86	0.90	0.591*
Pulmonology	0.3	0.02	<0.001*
General surgery	4.46	4.64	0.254*
Orthopedics	3.53	3.01	<0.001*
Ophthalmology	2.89	1.57	<0.001*
Otolaryngology	1.64	1.26	<0.001*
Thoracic surgery	0.55	0.72	0.006*
Neurosurgery	0.76	0.66	0.105*
Obstetrics and gynecology	2.07	1.85	0.035*
Cardiovascular surgery	0.72	0.77	0.405*
Urology	0.68	0.85	0.006*
Plastic surgery	0.07	0.04	0.134*
Dermatology	0.2	0.05	<0.001*
Interventional radiology	0.03	0.04	0.319*
Forensic science	0.01	0.00	0.223*
Psychiatry	0.08	0.02	0.002*

## Discussion

In this study, a significant decrease was detected in the emergency visits of all triage levels and emergency consultations. In our study, it was found that the rates of examinations and consultation of patients with red and yellow triage levels increased significantly. In this situation, we can say that the critical patients presented to the ED more than non-critical patients during COVID-19 outbreak.

In a multicenter study by Hoyer et al., they found a reduction in stroke and transient ischemic attack cases in some centers [9]. In the study conducted by Mafham et al., a decrease in all acute coronary syndrome types was found during the COVID-19 period [5]. Lange et al. stated in their report that during the COVID-19 period, hospital admissions decreased in life-threatening situations [4]. Similarly, in our study, a significant decrease was detected in the visits of critically ill patients at the red triage level and the consultations from the red area. Although there is a significant decrease in the count of critical patients, the rate of critical patients has increased within the total emergency visits in our study. Also, emergency consultation rates of critically ill patients have increased within the total emergency consultations. Nationwide information and clarification are needed for the public. It should be emphasized that non-COVID patients are also provided medical care in appropriate areas during the pandemic period. Additionally, appropriate triage scores could use to select the critical patients who need more resources and medical care [10].

A significant decrease was detected in the count of visits and visit rates at the green triage level. In this case, it can be argued that non-emergency patient visits decreased significantly during the COVID-19 period. Although the count of LWBS decreased, an increase in the LWBS rate was detected. The reason for this proportional increase may be the potential risk of contact with COVID-19 patients. It can be said that the count of non-emergency visits decreased during the pandemic period, and they tended to leave the ED before the examination at a higher rate.

When the consulted departments were evaluated, there was a significant decrease in consultations requested from most departments, while an approximately nine-fold increase in infectious disease consultations. This increase can be considered as a natural consequence of the COVID-19 outbreak.

COVID-19 cases have been reported in a broad clinical spectrum, from mild to severe conditions [11]. It has been reported that COVID-19 can cause severe diseases and the need for intensive care admission, especially in elderly patients [11]. The increase of critical patients due to COVID-19 infection also affected the count of anesthesiology consultations in our study. Although there was no significant difference in anesthesiology consultations in our study, it was found that the anesthesiology consultation rates reached approximately two times higher compared to the pre-pandemic period. The reason for this increase could be the proportional increase of COVID-19 critical patients in the red triage level within the total ED visits.

Toniolo et al. stated that severe cardiac cases decreased during the pandemic period [12]. Similarly, a significant decrease was found in the patients' count consulted to the cardiology department in our study. However, a significant increase in cardiology consultation rates was detected. This increase can be accepted due to the increase in the proportion of patients with red triage level and the higher need for the cardiac evaluation of the critical patients. Besides, the cardiac effects of both the COVID-19 virus itself and the drugs used for treatment may have caused an increase in cardiology consultation rates. A more decrease was detected in the count of patients consulted to the neurology department than the cardiology department consultations. Schwarz et al. also found a reduction in acute coronary syndrome and cerebrovascular disease cases similar to our study [13]. The reservation of some of the neurology and coronary intensive care beds in our hospital for COVID-19 intensive care patients has decreased the count of patients with neurological and cardiac diagnoses brought to the ED by the emergency medical service. As a result of this situation, the count of patients consulted in both departments has decreased.

Gastrointestinal and internal problems associated with COVID-19 have been reported in the literature [14, 15]. While the count of internal medicine and gastroenterology consultations decreased, the consultation rates did not change. COVID-19-related gastrointestinal and internal pathological conditions can be shown as the reason for similar consultation rates.

The studies conducted by Mitkovic et al. and Pichard et al. concluded that total trauma patients, hand and upper extremity injuries decreased during the COVID-19 outbreak [8, 16]. Patel et al. and Cano-Valderrama et al. found decreased surgical emergencies during the pandemic period [17, 18]. Similarly, in our study, a significant reduction was found in the count of visits to the red and yellow areas where surgical emergencies and trauma patients were presented. A similarly significant decrease was found in patients' count consulted to the general surgery and orthopedics departments. Similar to the reduction of the count of orthopedics consultations, the consultation rates within total consultations were decreased too.

The general surgery department was the most consulted clinic in the pre-pandemic period, and it was also the second most consulted clinic during the pandemic period. General surgery consultation counts decreased significantly during the pandemic period. The decrease in total ED visits may cause this situation. However, there was no significant change in general surgery consultation rates. Although the total count of neurosurgical consultations decreased, it increased proportionally. Also, there was no significant change in the count of thoracic surgery consultations during the pandemic period, but the thoracic surgery consultation rates increased. Our hospital continued to serve as a tertiary healthcare institution and a trauma center during the pandemic. Besides, ours is the only hospital in our region with a full-time thoracic surgeon on staff. These situations could cause the absence of significant decreases in critical patients’ proportional values and some of the departments’ emergency consultations requested from ED.

In a multicenter study conducted in Spain, researchers found an approximately 60% decrease in emergency surgery cases and an approximately 50% prolongation between the onset of symptoms and the presentation to the ED during the pandemic period [18]. The reasons for this change may be the restrictions and the fear experienced by the patients in many countries as in our country. Although there are restrictions across the country, our country’s emergency medical service has continued to serve patients with its increased capacity. The emergency call center has also helped emergency patients who have demanded to reach hospitals with their means by issuing permits during the restriction period. Therefore, fear plays a more significant role rather than the restrictions on the decrease in patient presentations. The reduction in all triage level visits in our study may also be due to fear. Also, Mantica et al. stated that an increasing COVID-19 daily mortality might affect ED admissions [19].

A moderate-strong correlation was found between the total examination count and all triage level patients and LWBS patients in our study. This relationship can be shown as the reason for a similar decrease in emergency visits and emergency consultations.

Similarly to Babu et al.’s study, the ophthalmology consultation counts and consultation rates were decreased during the pandemic period in our study [20]. The reason for this decrease may be to protect the social distance between patients and physicians because the ophthalmology examinations were face to face and closer than other examinations.

Limitations

The first limitation of our study is a single-center study. Since the data on the count of emergency surgery and cardiac interventions performed during the pandemic period were not included in the study, no discussion could be made on this issue. Since the demographic information, clinical follow-up processes, and clinical outcomes of the patients were not included in the study, the relationship between the count of visits and consultations could not be evaluated. No comments have been made regarding the small number of consultation requests in the pre-pandemic period.

## Conclusions

During the COVID-19 pandemic, emergency visits at all triage levels decreased. While the rate of critical patient visits increased, non-emergency visits decreased. The total count of consultations requested from the emergency department decreased. Anesthesiology and cardiology consultation rates increased; neurology, orthopedics, and ophthalmology consultation rates decreased; and internal medicine and general surgery consultation rates did not change within total emergency consultations. Necessary measures should be taken, and public information should be provided to ensure that all patients can safely present to hospitals and be evaluated, especially non-COVID critical patients.

## Appendices

**Table 5:** Example cases according to triage levels

Triage Level	EXAMPLE CASES
Green	All kinds of simple symptoms in the patient whose general condition and vital signs are stable
	Simple wounds - minor abrasions, simple incisions that do not require stitches
	All kinds of pain that are not high risk and are mild
	Low-risk disease history without active complaints
	Behavioral and psychological disorders with chronic symptoms and good general condition
Yellow	High blood pressure with Diastolic > 110 mmHg, Systolic > 180 mmHg
	Moderate blood loss for any reason
	Moderate respiratory distress in which auxiliary respiratory muscles do not participate in breathing
	Seizure history (awake)
	Oncology patient with fever or a patient using steroids
	Stubborn vomiting
	Conscious patient with head injury with amnesia
	Chest pain inconsistent with cardiac history
	Patient over the age of 65 with abdominal pain
	Patient with severe abdominal pain
	Limb injury including deformity, severe laceration, and crush injury
	A child at risk or suspected of abuse
	Stressful and self-harming patient
	Simple bleeding
	Simple chest injuries without chest pain and respiratory distress
	Swallowing difficulty without respiratory distress
	Minor head injuries without loss of consciousness
	Vomiting and diarrhea without signs of dehydration
	Eye inflammation or foreign body with normal visual function
	Minor extremity trauma (ankle sprain, possible simple fracture, uncomplicated laceration requiring investigation) normal vital signs
	Non-severe abdominal pain
	Patients with behavioral disorders without risk of harm
Red	Cardiac arrest
	Respiratory arrest
	Risk of airway obstruction
	Major multiple trauma
	Respiratory rate < 10/minute
	Systolic Blood Pressure < 80 mmHg (adults) or children or infants whose general condition is impaired
	Ongoing or prolonged seizure
	Unresponsive or hypoventilation in the patient with drug overuse
	Chest pain similar to cardiac pain
	Patients with severe shortness of breath in which the auxiliary respiratory muscles participate in respiration or whose pulse oximetry value is <90% if it can be observed.
	Risk of airway obstruction with severe stridor or difficulty swallowing
	Circulatory disorder
	Moist, cold skin, perfusion disorder
	Heart rate <50 or > 150 per minute
	Hypotension with hemodynamic findings
	Acute hemiparesis / dysphasia
	Fever with lethargy (all ages)
	Eye contact with acid/alkali that requires irrigation
	Severe localized trauma such as major fracture or amputation
	Severe pain for any reason
	Oral intake of significant sedatives or other toxic substances
	Violent, aggressive behavior
	Behavior that harms oneself or others
